# Identification of Topological Network Modules in Perturbed Protein Interaction Networks

**DOI:** 10.1038/srep43845

**Published:** 2017-03-08

**Authors:** Mihaela E. Sardiu, Joshua M. Gilmore, Brad Groppe, Laurence Florens, Michael P. Washburn

**Affiliations:** 1 Stowers Institute for Medical Research, Kansas City, MO 64110, USA; 2 Department of Pathology and Laboratory Medicine, The University of Kansas Medical Center, 3901 Rainbow Boulevard, Kansas City, Kansas 66160, USA

## Abstract

Biological networks consist of functional modules, however detecting and characterizing such modules in networks remains challenging. Perturbing networks is one strategy for identifying modules. Here we used an advanced mathematical approach named topological data analysis (TDA) to interrogate two perturbed networks. In one, we disrupted the *S. cerevisiae* INO80 protein interaction network by isolating complexes after protein complex components were deleted from the genome. In the second, we reanalyzed previously published data demonstrating the disruption of the human Sin3 network with a histone deacetylase inhibitor. Here we show that disrupted networks contained topological network modules (TNMs) with shared properties that mapped onto distinct locations in networks. We define TMNs as proteins that occupy close network positions depending on their coordinates in a topological space. TNMs provide new insight into networks by capturing proteins from different categories including proteins within a complex, proteins with shared biological functions, and proteins disrupted across networks.

Protein interaction networks are dynamic systems. They differ depending on cellular context and under different conditions. Perturbing protein complexes and protein interaction networks is a valuable way to study protein complex dynamics. For example, in *S. cerevisiae*, one effective way to perturb a protein interaction network is to study protein complexes in genetic deletion backgrounds^1^,^2^,^3^,^4^. In a recent study of mammalian protein complexes, the authors found variable members of complexes in different cell types and suggest paralogue switching as an important mechanism of protein complex control^5^. In diseases like cancer, altered networks due to mutation is an area of active study^6^. In one study, an analysis of a mutant EGFR interactome in lung cancer cell lines facilitated the identification of compounds that could overcome drug resistance^7^. A recent large scale analysis found widespread disruption of protein interactions by human disease-associated missense mutations^8^. Differential protein interaction network analysis leads to new insights into biology^9^, and methods continue to be developed to facilitate such analyses^10^.

One major challenge in the study of perturbed protein interaction networks is how to visualize and analyze this data in order to gain deeper insights into the organizational principles of such networks. One promising and emerging approach for analyzing large scale datasets is topological data analysis (TDA)^11^. TDA functions as a geometric approach for analyzing multidimensional complex data and to identify key features of the data which may not be apparent with traditional methods. TDA has been successfully used in very diverse areas of research like gene expression profiling on breast tumors^12^, identification of different types of diabetes^13^, viral evolution^14^, spinal cord and brain injury^15^, disease response to pathogens^16^, human recombination^17^ voting behavior of the members of the US House of Representatives^11^, and characteristics of NBA basketball players via their performance^11^. We have used TDA to study the conservation of human and yeast chromatin remodeling networks^18^ and the associations of the uncharacterized WDR76 protein with DNA damage and chromatin remodeling proteins^19^.

In this body of work, we investigated the capabilities of TDA for the analysis of perturbed protein interaction networks from two different species. First, we generated a deletion network dataset of the INO80 complex in *S. cerevisiae*. INO80 is a conserved protein complex with important biological roles in transcription, chromatin structure, DNA replication and DNA repair^20^,^21^,^22^,^23^. For the analysis of the INO80 perturbed network, we affinity purified wild-type protein complexes and compared them to the affinity purification of protein complexes when certain components of the INO80 complex were deleted from the strain analyzed. We next reanalyzed a human histone deacetylase (HDAC) protein interaction network centered on the Sin3 complex that was perturbed with the HDAC inhibitor suberoylanilide hydroxaminic acid (SAHA)^24^. SAHA is an important human therapeutic having been approved for treatment of patients with progressive, persistent, or recurrent cutaneous T-cell lymphoma^25^,^26^, and SAHA is also the subject of many additional cancer clinical trials^27^. TDA greatly facilitated the organization of associated proteins in clusters and created a novel visual representation of the interaction networks. In our analysis of both networks we were able to rapidly identify biologically relevant modules using TDA. Surprisingly, these modules could contain proteins with different features like proteins in a complex, proteins with distinct biological functions, or proteins altered by the system. Capturing these classes of proteins would typically require multiple different computational approaches asking a specific question. Here, we term these modules Topological Network Modules (TNMs), which are proteins that occupy close network positions depending on their coordinates in a topological space.

## Results

### Quantitative Proteomic Analysis of the Disrupted S. cerevisiae INO80 Complex

**Figure 1:**
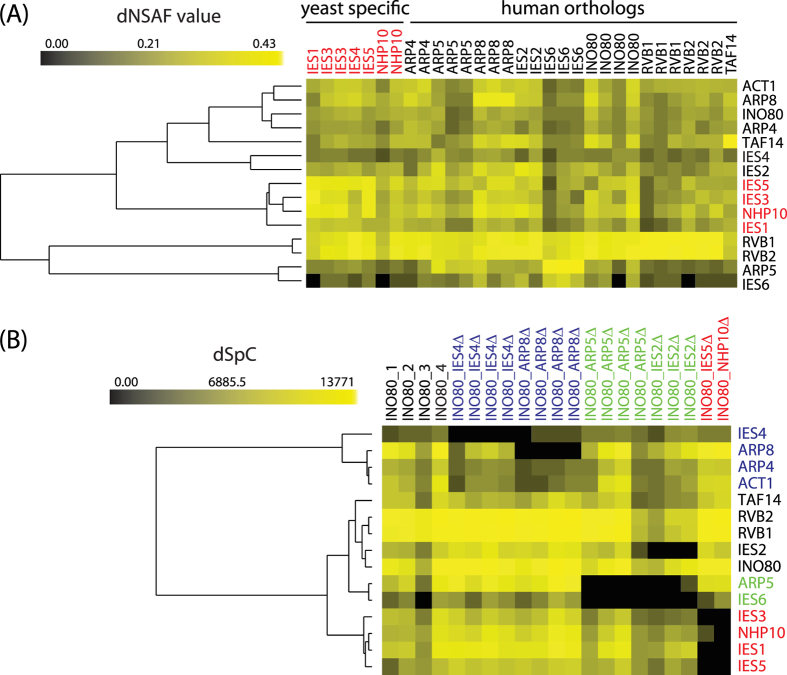
Hierarchical clustering of INO80 complexes in wild type and genetic deletion backgrounds. (**A**) A total of 14 different subunits of the Ino80 complex were used as baits for the TAP purification and then analyzed by MudPIT and dNSAF label-free quantitation. Hierarchical clustering of the 31 total analyses is shown with dNSAF values as input. Proteins that are yeast specific are in red, while proteins that are orthologous to human proteins are in black. (**B**) The INO80 complexes from seven different deletion strains were purified using Ino80 as the TAP-tagged subunit and analyzed by MudPIT. Four biological replicates of wild type *INO80-TAP* are clustered with four biological replicates of *INO80-TAP IES4Δ*, four biological replicates of *INO80-TAP ARP8Δ,* four biological replicates of *INO80-TAP ARP5Δ*, three biological replicates of *INO80-TAP IES2Δ*, one analysis of *INO80-TAP IES5Δ*, and one analysis of *INO80-TAP NHP10Δ*. The proteins belonging to the ARP8, ARP5, and NHP10 modules are in blue, green and red, respectively, while proteins that were not significantly altered by the mutants are in black. In both (**A**) and (**B**), the color intensity represents protein abundance with bright yellow displaying highest abundance and black indicating that the protein was not detected in a particular purification.

To begin our investigation into the topology of perturbed protein interaction networks, we first carried out a quantitative proteomic analysis of the *S. cerevisiae* INO80 protein interaction network. Yeast INO80 consists of ten evolutionarily conserved subunits orthologous to the human INO80 complex and five yeast specific subunits^22^,^23^. Here, we affinity purified the proteins associated with all five yeast specific subunits and nine of the ten conserved subunits. Replicates were also performed in our analysis which resulted in a total of 31 purifications (Supplementary Table S1A). Identification of proteins was accomplished using multidimensional protein identification technology (MudPIT)^28^. The relative abundance of proteins was obtained from spectral counts and are represented by dNSAF values^29^. All 15 subunits of the INO80 complex were identified in a reproducible manner in these affinity purifications (Supplementary Figure S1A–O and Supplementary Table S1A). Then, we applied hierarchical clustering^30^ on the core proteins using their relative abundance in order to investigate the possible organizational interactions generated from wild-type, i.e. unperturbed, affinity purifications (Fig. 1A). Four yeast specific subunits (IES5, IES3, IES1, and NHP10) were clustered together in a separate group apart from the orthologous proteins indicating that yeast specific proteins form a module within the INO80 complex (Fig. 1A). However, the orthologous proteins were dispersed throughout the hierarchical dendogram.

Next, we analyzed the complex using a genetic deletion approach that we have previously used to study the modular architecture of the Rpd3^4^ and SAGA networks in *S. cerevisiae*^3^. In this approach, we analyzed protein interactions of complexes after deleting individual genes in a complex from the genome of *S. cerevisiae*. Here, we deleted six subunits positioned in different possible modules of the INO80 complex and purified the resulting complexes via the TAP-tagged Ino80, Arp8, Ies2 or Ies6 subunits. Specifically, we analyzed eleven deletion strains, namely *INO80-TAP IES4Δ, INO80-TAP ARP8Δ, INO80-TAP ARP5Δ, INO80-TAP IES2Δ, INO80-TAP IES5Δ, INO80-TAP NHP10Δ, ARP8-TAP ARP5Δ, ARP8-TAP IES2Δ, IES2-TAP IES4Δ, IES2-TAP ARP8Δ*, and *IES6-TAP IES4Δ* (Supplementary Table S1B). Including replicates, a total of 23 affinity purifications from deletion strains were analyzed. The relative abundance, as estimated by dNSAF values, and reproducibility of detection observed for the 15 INO80 subunits in each of the deletion strains were compared to the corresponding wild-type affinity purifications (Supplementary Figure S1A–K).

Hierarchical clustering of the deletion network derived from the six perturbed complexes purified via *INO80-TAP* revealed three modular patterns (Fig. 1B). Deletions of the IES4 or ARP8 genes both resulted in the loss or significant decrease in the recovery of each other, as well as of the Arp4 and Act1 subunits (Fig. 1B and Supplementary Figure S1A/D), hence defining the Arp8 module. Similarly, complexes purified from the *IES2-TAP IES4Δ, IES6-TAP IES4Δ* and IES2*-TAP ARP8Δ* strains were affected in the detection of the Arp8 module components (Supplementary Figure S1B/C/E). Next, the Arp5 module was defined as containing Arp5 and Ies6 since deletion of ARP5 led to the loss of the Ies6 subunit (Fig. 1B and Supplementary Figure S1H). Additional analyses conducted on *ARP8-TAP ARP5Δ* showed that Arp5 and Ies6 were both lost from the complex (Supplementary Figure S1I), adding validation to the identity of the Arp5 module. Both of these subunits were also lost when the IES2 gene was deleted (Fig. 1B and Supplementary Figure S1F–G) suggesting that Ies2 likely brings the Arp5 module to the larger Ino80 complex. Finally, deleting NHP10 and IES5 (Fig. 1B and Supplementary Figure S1JK) resulted in the loss or significant decrease in the levels of the *S. cerevisiae* specific Nhp10, Ies1, Ies3 and Ies5 components, hence defining a third structural module.

In addition to these three modules, we also noticed another group of proteins (Taf14, Rvb1/2, Ies2, and Ino80) that were not severely altered by any of the deletions (all five subunits are present in all purifications; Fig. 1B and Supplementary Figure S1A–O), indicating that it is appropriate to treat them as interacting proteins outside of these modules. This result can be explained by the fact that three of these proteins (Taf14, Rvb1 and Rvb2) are also associated with at least one complex outside of the INO80 complex. For example, Rvb1 and Rvb2 are shared by at least three complexes (INO80, NuA4, and SWR) and Taf14 is also a component of several DNA-interacting complexes. Thus, these proteins are modular by definition to be accessible to other complexes. In the case of the Ino80 protein, a structural study revealed its potential role as a scaffold protein^31^. Overall, our analysis of the deletion network and the modularity of the *S. cerevisiae* INO80 complex were in agreement with prior structural and biochemical analyses^31^,^32^,^33^.

### Identifying Topological Modules in the INO80 Deletion Network

**Figure 2:**
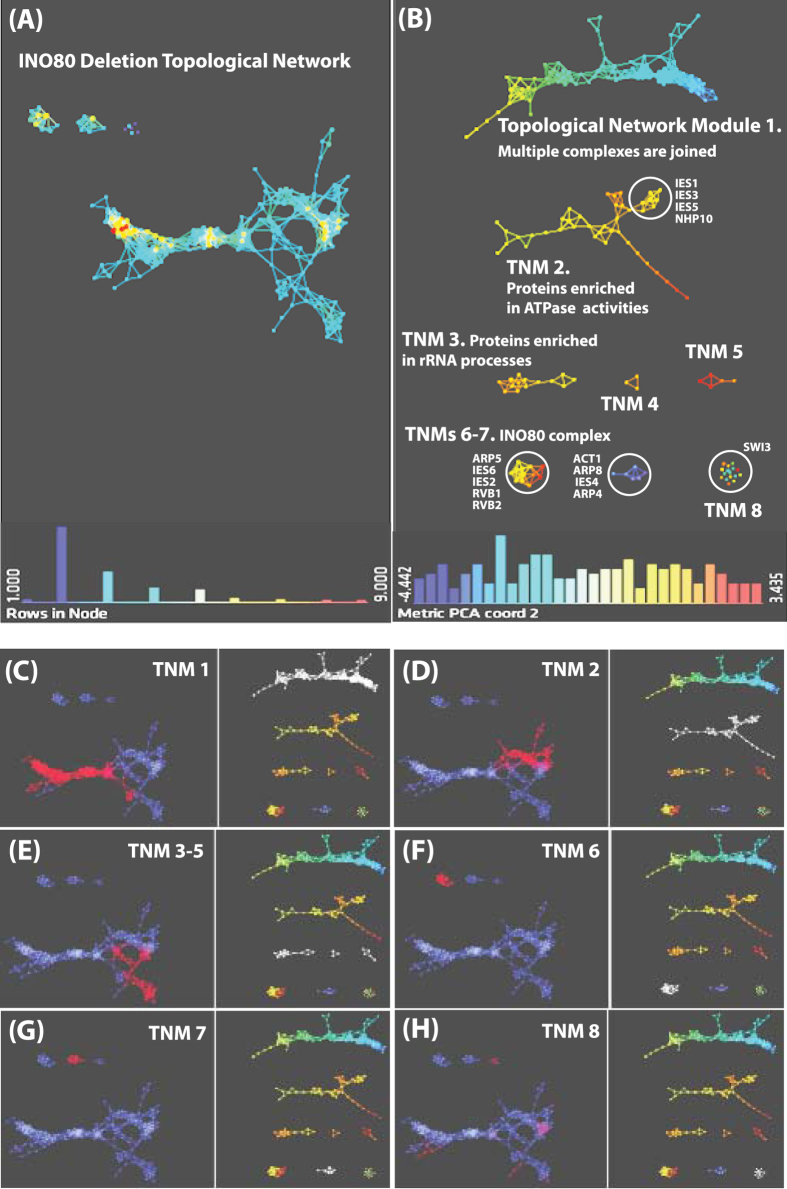
Topological Data Analysis of the INO80 deletion network. (**A**) TDA was used to analyze the fold-change ratios of 149 proteins detected from the AP-MS analysis of the INO80 deletion strains. Panel (A) represents a complete view of the network, while on the right side (**B**) the network is separated into eight modules. Filters with correlation metric were used such as resolution and gain were set at 30 and 3.0x eq. in (**A**), and 45 and 3.0x eq. in (**B**). (**C**,**H**) Individual TNMs, highlighted in white in the corresponding right panel, are mapped onto the main network, highlighted in red in the corresponding left panel. TNMs were numbered in order to emphasize their locations within the topological network structure. In (**A**–**H**), protein nodes are colored based on the metric PCA2. Color bar: red: high values, blue: low values. Node size is proportional to the number of proteins in the node.

As we have shown previously, Topological Data Analysis (TDA) has proven useful to rapidly organize and mine affinity purification datasets^18^,^19^, which led us to investigate what insights it could provide with a deletion network dataset. Proteomics datasets generated from the analysis of INO80 wild-type and genetic deletions were used to construct a TDA network of the extended INO80 disrupted interactions. In this case, all of the proteins detected in our samples were considered for the statistical analysis, not just the core components of the INO80 complex. First, wild-type data was compared against a negative control dataset to ensure that non-specific proteins were not included in the analysis (Supplementary Table S2A). Second, QSPEC^34^ was used on this filtered protein list to calculate fold change ratios between spectral counts measured in wild-type and genetic deletions and determine significant changes in protein levels between these two datasets (Supplementary Table S2B). We retained only proteins that had a significant QSPEC log2 fold-change of −2 or less, which corresponds to a fold change of 4 or higher, in at least one of the mutants. The final group of 196 proteins passing this criteria comprised the subunits of the INO80 complex and proteins outside the complex (Supplementary Table S2C). We then subjected these proteins to TDA^11^ to determine the spatial positions of protein nodes and build a perturbed topological network (Fig. 2A).

We next asked what the shared features of proteins in this network were. Resolution and gain settings were selected to break the network into distinct modules (Fig. 2B). By exploring geometric relationships in a topological manner, we discovered biologically meaningful information. As shown in Fig. 2B, eight large groupings of protein nodes that we termed topological network modules (TNMs) were identified with distinct characteristics. For example, TNM 1 contained information where multiple complexes were joined and TNM 2 contained proteins enriched in GTPase and ATPase activities and the components of the yeast specific NHP10 module (Ies1, Ies3, Ies5, and NHP10). In contrast to TNMs 1 and 2, we identified isolated nodes as well as completely disconnected nodes in TNM 8 (Fig. 2B). TNMs 6 and 7 are two of the isolated nodes consisting of subunits of the ARP5 and ARP8 structural modules, respectively (Fig. 2B). We observed that when we deleted subunits of the ARP5 module only a small number of proteins were altered, mostly subunits of the ARP5 module, and hence these nodes were connected outside the main TDA structure. However, in addition to the ARP5 module, two known interacting partners, Eno2 and Fba1^35^, were also identified in close space with the ARP5 module. Interestingly, Rvb1 and Rvb2 are located in close proximity to the subunits of the ARP5 module, showing agreement with the results obtained from Tossi *et al*.^31^. This shows that our topological method properly identifies the correct structural modules with the INO80 complex. Furthermore, this network structure can highlight nodes of higher or lesser connections and suggests how distinct biological complexes are joined. For example, we could see that modules with larger number of nodes are more central in the network.

We next sought to map where each of these individual modules was located on the full topological network shown in Fig. 2A. To do so, we superimposed each module onto the complete topological network. Remarkably, each module mapped to a distinct location in the complete topological network. TNM 1 was located at the tail end of the network (Fig. 2C), followed by TNM 2 which was located in the upper flare of the network (Fig. 2D). TNMs 3–5 were positioned on the lower flare of the network (Fig. 2E). TNMs 6 and 7 were located outside the main network (Fig. 2F,G), and TNM 8 consisted of disconnected proteins that were distributed throughout the network (Fig. 2H). TNM 8 is particularly interesting since it consisted of proteins that were altered in most mutants. For example, two components of the SWI/SNF complex, Snf12 and Swi3, were proteins that showed a significant change in all the mutants (TNM 8, Supplementary Table S3), demonstrating a link between the INO80 and SWI/SNF complexes.

**Figure 3:**
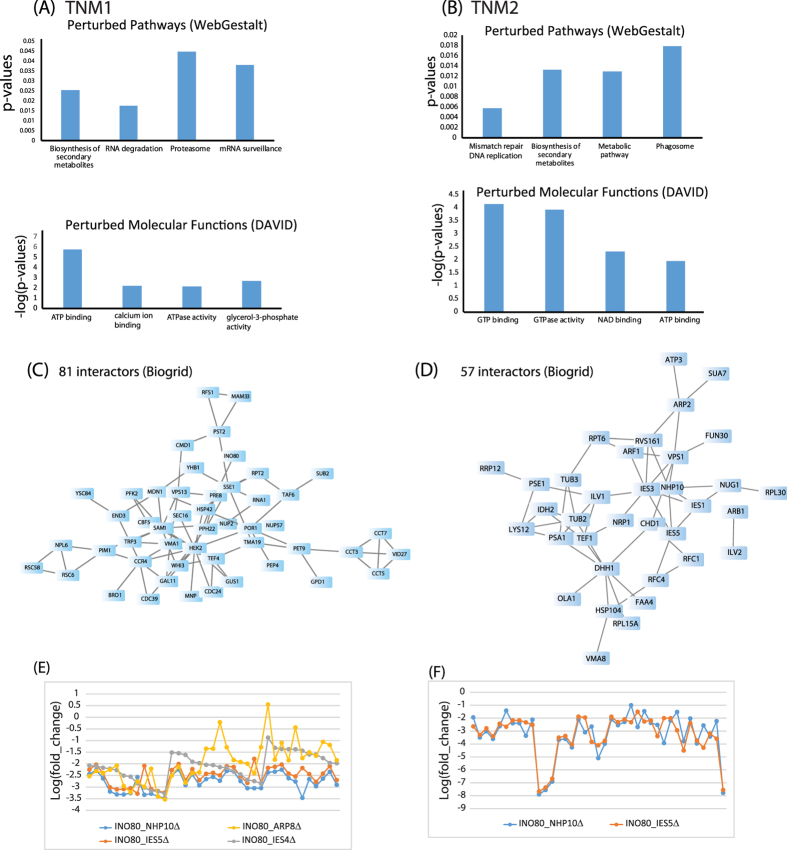
Overall model of the INO80 deletion network. Enrichment Analysis (**A**) and (**B**) Altered protein interactions in TNMs 1 and 2 were searched for biological pathways and molecular functions enrichment using WebGestalt^36^ and DAVID^37^ annotation tools. The top 4 enriched terms in biological pathways and MF as indicated by their significant p-values are illustrated in (**A**) and (**B**). (**C**) and (**D**) Network visualization of the overlap interactions within TMNs 1 (**C**) and 2 (**D**) and Biogrid database^38^. The protein networks were built using Cytoscape^51^. Changes in abundance of proteins within TNMs 1 and 2. (**E**) Proteins in TNM1 were plotted using their corresponding fold-ratios in the four mutants of the NHP10 and ARP8 modules. (**F**) Proteins in TNM2 were plotted using their corresponding fold-ratios in the two mutants of the NHP10 module.

We performed pathway analysis using WebGestalt^36^, and molecular function enrichment analysis using DAVID^37^ annotation tools, on the altered proteins in TNMs 1 and 2. The analyses revealed notable pathways and molecular function alteration (Fig. 3A,B). Pathways perturbed in TNM 1 were related to mRNA surveillance and the proteasome, for example (Fig. 3A). In contrast, pathways perturbed in TNM 2 were related to the phagosome, for example (Fig. 3B). Both modules show significant connection to the biosynthesis of secondary metabolites pathway (Fig. 3A,B). A closer look at the molecular function enrichment revealed ATP binding, ATPase activity, GTP binding and GTPase activity were in particularly enriched within modules (Fig. 3A,B). Next, we assessed the interactions in these two modules searching for overlaps with interactions in the Biogrid database^38^. We found 81 interactions between proteins in the TNM 1 and 57 interactions in the TNM 2 suggesting that these proteins are important elements for these functional relevant classes (Fig. 3C and D). Taken together these results suggest that protein complexes, pathways and protein interactions between these modules tend to be unstable in response to perturbation of the INO80 protein interaction network.

TNMs 1 and 2 contained the most proteins and were analyzed using ConsensusPathDB^39^ to determine the enrichment of protein complexes. TNM 1 showed enrichment for several complexes such as RSC (p-value = 0.0019, q-value = 0.004064), T- complex (p-value = 0.000207, q-value = 0.004064), and CCR-NOT (p-value = 0.008 and q-value = 0.0103). TNM 2 showed an enrichment for the RFC heteropentamer complex (p-value = 0.0014, q-value = 0.00279). TNMs 1 and 2 were further analyzed using GeneMANIA^40^ to examine their biological significance. TNM 1 showed enrichment for several complexes such as RSC (FDR: 4.33e-18), T- complex (FDR: 4.65e-9), and CCR-NOT (FDR: 9.32e-5). This group of proteins in TNM 1 were altered by mutants corresponding to the ARP8 and NHP10 modules of INO80 (Fig. 3E), suggesting possible shared biological function of these protein complexes with the two structural modules. TNM 2 showed a strong enrichment (FDR: 5.39e-10) for ATPase activity. This group of proteins in TNM2 displayed a significant change in the mutants corresponding to the NHP10 module (Fig. 3F). The NHP10 structural module itself was identified in this group, showing agreement with the topological result.

**Figure 4:**
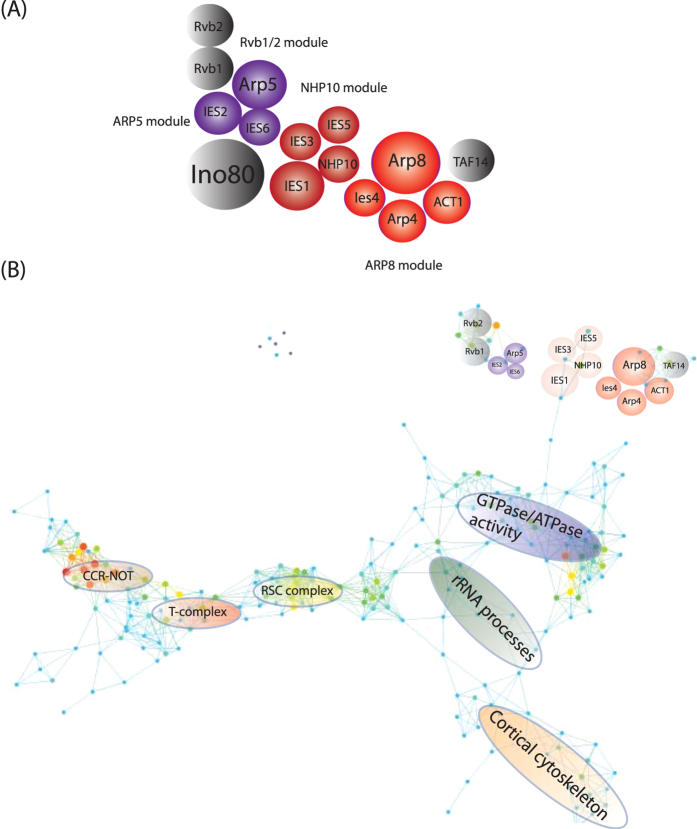
Construction of a low-dimensional structure of the INO80 complex. (**A**) Proteins were assembled based on the clustering results. Here we illustrated the relationship between proteins in the ARP5 and ARP8 modules and the display the final assembled complex. Red corresponds to the proteins in the NHP10 submodule, blue corresponds to the proteins in the ARP8 module and the ARP5 module was colored in green. Proteins that were not significantly altered by the mutants are colored in grey. The size of the inset circles corresponds to the molecular weights of the proteins illustrated. (**B**) Ino80 structural modules were mapped onto the overall network module generated by TDA. In addition, the localization of biological functions provided by GeneMANIA^40^ was mapped onto their general location of the TDA network.

On the basis of these findings we constructed a map of the structural modules within the INO80 complex (Fig. 4A). The composition of the INO80 complex corresponds to the structural modularity of the INO80 complex analyzed using cryo-electron microscopy^31^. However, cryo-electron microscopy does not provide insights into the larger network beyond the core protein complex. Here, we built a model of the complete topological network with the modules of this network and biological functions mapped onto the network (Fig. 4B). The INO80 structural submodules and the localization of GeneMANIA biological functions were mapped onto their general location in the network (Fig. 4B). This represents a new way of visualizing a perturbed protein interaction network.

Our analysis suggests the presence of topological network modules (TNMs) that are distinct from the standard definition of a module, which would be a group of proteins within an individual complex. TNMs are proteins that occupy close network positions depending on their coordinates in a topological space. TNMs can capture proteins from several different contexts. For example, TNMs can contain proteins from a complex (like TNM 6), proteins enriched for a shared biological function (like TNM 2), or proteins outside the complex disrupted in the deletion network (like TNM 8). In contrast to GO analysis, for example, TDA is hence capable of capturing multiple distinct features within a network.

### Comparison to Other Clustering Methods

We next sought to replicate the results performed through TDA analysis by applying two wildly used clustering approaches. K-means and hierarchical clustering analyses were performed on the QSPEC^34^ ratios. To a large extent, both methods replicate the submodules of the INO80 complex, however the rest of the modules were not significantly superimposable with those of TDA (Supplementary Figures S3 and S4 and Supplementary Table S4). To emphasize this discrepancy, we focused on TNM 2 where most of the proteins have a significant fold change between wild-type and INO80-TAP NHP10Δ and ARP8-TAP ARP5Δ genetic mutants. Thus these altered proteins should be located near the NHP10 submodule. Only 14 proteins overlapped with cluster 2 (i.e. this cluster has the largest overlap) generated by the K-means method (Supplementary Table S4). Proteins in TNM 2 were also spread out in the resulted hierarchical clustering (Supplementary Figure S3). This shows that separating proteins in large AP-MS data using single-dimensional space is still representing a challenge. Conventional algorithms such as hierarchical clustering or k-means are not ideal on large datasets where hundreds of prey proteins are associated with a much smaller number of samples. Furthermore, the organized TNMs are easier to interpret, the method automatically chooses an optimal number of clusters, prey proteins are assigned to single or multiple clusters and the connection between clusters can be determined. For this reason, TDA is a valuable tool for analyzing and visualizing large amount of data. Given that large data will continue to be generated, automatic procedures are needed to visualize and organize these data sets and avoid subjective intervention as much as possible.

### Topological Network Modules in a Human Drug Network

To further investigate the existence of TNMs in protein interaction networks, we reanalyzed a human histone deacetylase (HDAC) network centered on the Sin3 complex perturbed with the HDAC inhibitor suberoylanilide hydroxaminic acid (SAHA)^24^. In 2006, SAHA (Vorinostat) was approved for treatment of patients with progressive, persistent, or recurrent cutaneous T-cell lymphoma^25^,^26^, and SAHA is also the subject of many additional cancer clinical trials^27^. We and others have studied the effect of SAHA on human Sin3 complexes to elucidate the mechanism of action of this drug beyond the simple inhibition of HDAC activity^24^,^41^,^42^.

**Figure 5:**
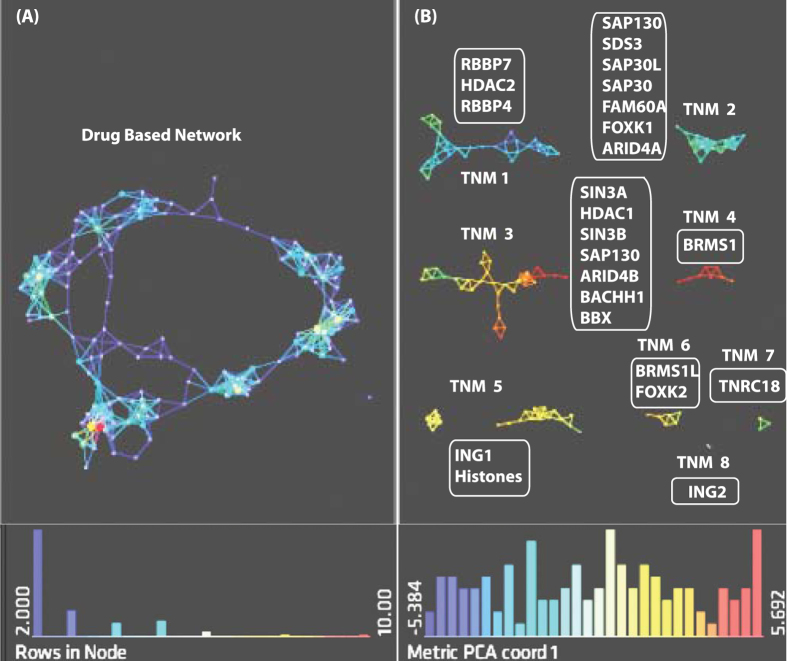
Topological Data Analysis of the SAHA-perturbed Sin3 network. A reanalysis of the effect of SAHA on six affinity purifications from Sardiu *et al*.^24^ was conducted. Z-scores from QSPEC^34^ were used to build a topological network (**A**). In (**B**), the description of the eight modules identified is shown with the list of proteins belonging to each TNM provided as Supplementary Table 6. Filters with norm correlation metric were used (resolution 20, gain 3.0x eq. in **A**), and resolution 30, gain 3.0x eq. (in **B**). Proteins are colored based on the metric PCA1. Color bar: red: high values, blue: low values. Node size is proportional with the number of proteins in the node.

We applied TDA to investigate the interactions when the Sin3 complex is exposed to SAHA by analyzing the six previously reported affinity purifications obtained from cells in the presence and absence of the drug. The proteins that demonstrated significant change between DMSO- and SAHA-treated samples are provided in Supplementary Table S5. Z-scores obtained from QSPEC^34^ were used in the construction of a topological network (Fig. 5A). Next, we selected resolution and gain settings to break apart the TDA network into TNMs (Fig. 5B). Subunits of the Sin3/HDAC complex were distributed in each of these eight identified TNMs. TNM 1 contained Rbbp4, Rbbp7 and Hdac2, which are core components of the complex, along with 25 additional proteins (Supplementary Table S6). TNM 2 contained many subunits of the Sin3 complex including Sin3A, Hdac1, Sin3B, Sap130, Arid4B, Bachh1, and Bbx, and proteins involved in DNA repair (Supplementary Table S6). TNM 2 contained proteins that were particularly affected by SAHA treatment of Sap30 and Sap30L affinity purified complexes^24^. This module includes the direct interaction between Sin3A and Hdac1^43^. TNM 3 included proteins that were affected by the drug when Brms1 and Brms1L were used as baits^24^, which were Sap130, Suds3, Sap30L, Sap30, Fam60A, Foxk1 and Arid4B (Supplementary Table S6). Brms1, Brms1L, Ing1 and Ing2 are each distributed in different TNMs (Supplementary Table S6), which is consistent with the observation that Brms1-Brms1L and Ing1-Ing2 are mutually exclusive pairs^24^. The isolation of Ing2 (Fig. 5B) from the rest of the subunits of the complex is in agreement with the observation that the recovery of this subunit is greatly reduced when all baits are exposed to SAHA drug and it shows that Ing2 is a critical component of the complex^24^,^42^.

**Figure 6:**
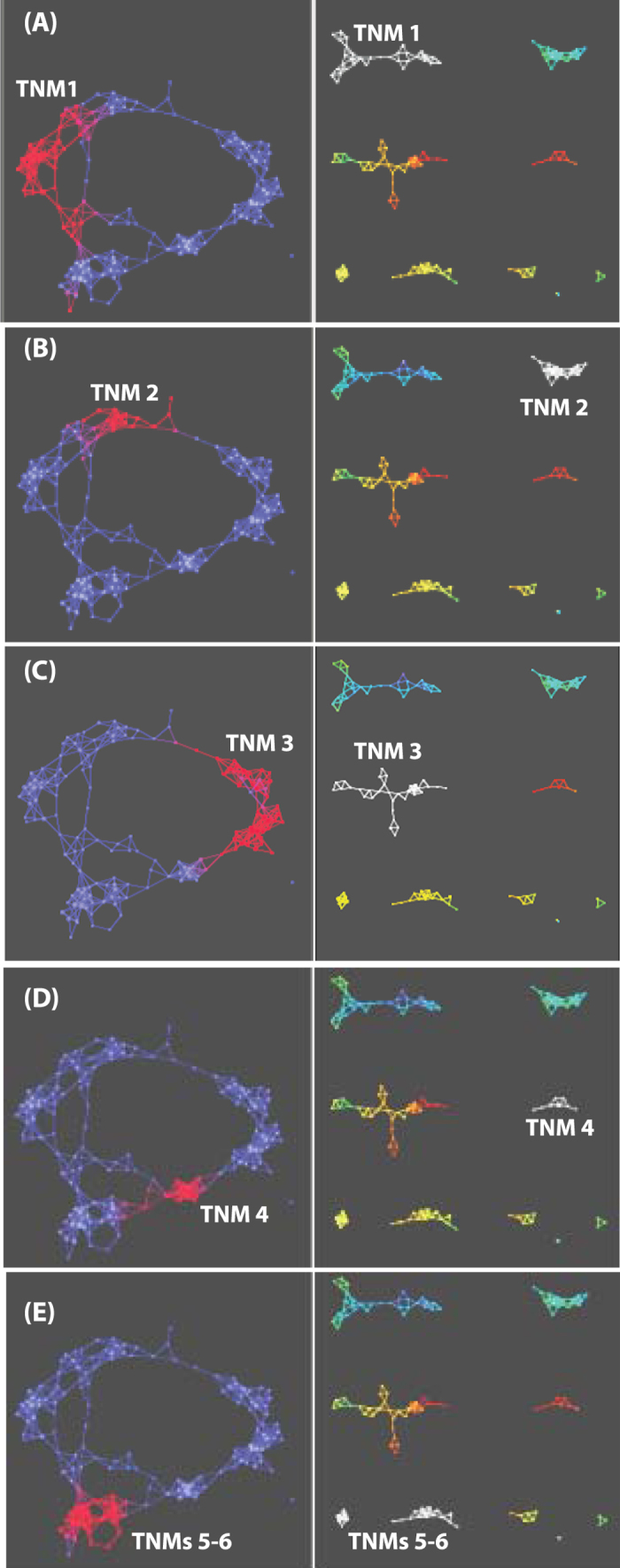
Topological Network Modules in the SAHA-perturbed Sin3 network. In a further analysis of the data presented in Fig. 5, individual topological network modules, highlighted in white in the right panels, are mapped onto the main network, highlighted in red in the left panels. In this figure we illustrate the location and the connection between TNM 1 (**A**), TNM 2 (**B**), TNM 3 (**C**), TNM 4 (**D**), and TNMs 5–6 (**E**) within the Sin3-drug network. Filters with norm correlation metric were used (resolution 30, gain 3.0x). Proteins are colored as in Fig. 5.

As with our analysis of our INO80 deletion network dataset, we next mapped the eight TNMs of the Sin3/SAHA network onto the full TDA network (Fig. 6). Again, we found that each module mapped onto a distinct location of the overall TDA network (Fig. 6A–E). However, in the Sin3/SAHA network there is a distinct circular pattern of TNMs compared to the INO80 network: each TNM is linked to at least one other TNM, but some TNMs are not connected to each other. For example, TNM 1 is linked to TNM 2 and TNM 5 but not to TNMs 3 and 4. Unlike in a hierarchical clustering, where there is no obvious relationship between clusters, here links that bridge TNMs could be examined in details offering a new way to exploit the topological feature of protein interaction networks.

## Discussion

Large scale datasets are increasingly generated in many disciplines. New and improved methods are continually needed to accelerate analysis of such large scale datasets and to generate new insights into the system being analyzed. In addition, network perturbation is an important tool to gain insights into the resiliency of a network, how information flows through a network, and what is the effect of a disruption on a network. However, the challenge remains regarding how to efficiently and effectively analyze such datasets. A google image search for a term like ‘network analysis’ reveals many images that are represented with large numbers of nodes that are connected by individual lines that then grow into large ‘hairball’ like representations. Such images and analyses can be useful in many disciplines for finding new connections in a network, however they lack the ability to provide deeper insights, for example, regarding how groups of nodes behave in a network.

The use of TDA to analyze very diverse network types, ranging from NBA basketball players^11^ to human recombination^17^, is growing and proving highly valuable^11^,^12^,^13^,^14^,^15^,^16^,^17^. We have previously used hierarchical clustering approaches to study protein interaction networks and perturbed protein interaction networks^3^,^4^,^30^,^44^. However, one significant weakness of these studies was the reduction of the dataset to focus on a limited number of components in an individual protein complex, rather than taking a broader view of all the proteins in the dataset. We have recently turned to TDA to facilitate our analyses of protein interaction network datasets by investigating the conservation of a chromatin remodeling network^18^ and to analyze the associations of a new protein involved in DNA damage^18^.

In this study, we used TDA to analyze perturbed protein interaction networks. First, we compared the data generated from an analysis of intact INO80 protein complexes compared to disrupted INO80 protein complexes in *S. cerevisiae*. Here, the disruption was the analysis of protein complexes where individual components of the complex were deleted from the *S. cerevisiae* strain. Using standard methods of analysis, we were able to determine the relative abundance of proteins in the complexes and how each disruption affected the complex. The modularity of the complex captured using this approach was in strong agreement with prior focused studies of the INO80 complex^31^. TDA revealed an overall network shape as a flare and individual modules mapped onto distinct portions of this flare. What was most revealing in this network was that TDA not only captured the structural modularity within the complex but also captured modularity in the entire network. We named such modules topological network modules (TNMs). The TNMs in the disrupted INO80 network captured proteins from different categories including proteins within a complex, proteins with shared biological functions, and proteins disrupted across networks. We next applied TDA to the analysis of a previously published perturbed human protein interaction network where the HDAC inhibitor SAHA was used to disrupt the human Sin3 protein interaction network^24^. In this case, TDA revealed a distinctly different shaped network than the INO80 network. Here the Sin3/SAHA network was a circular shape with distinct TNMs again mapping onto distinct portions of the network. However, in this circular shape TNMs were more clearly separated from each other.

There is growing interest in the importance of investigating network dynamics^9^. Reasons for this included the extensive disruptions in protein interactions by human genetic disorders^8^ with a clear interest in studying the effects of mutations on altered networks^6^. As more perturbed protein interaction networks become available, analyzing these datasets with advanced mathematical tools like Topological Data Analysis will likely provide new insights into these systems. We expect that topological network modules will be found in most if not all perturbed network analyses leading to new insights into network organization.

## Methods

### Affinity purifications and mass spectrometry

All yeast cells were grown in YPD to an absorbance of OD_600_ 1.5–2.0. TAP was performed as previously described^18^,^45^. To analyze the purified protein complexes, TCA-precipitation, LysC/Trypsin digestion, and multidimensional protein identification technology (MudPIT) analyses were performed as previously described^28^. RAW files were converted to the ms2 format using RAWDistiller v. 1.0, an in-house developed software. The ms2 files were subjected to database searching using SEQUEST (version 27 (rev.9))^46^. Tandem mass spectra of proteins purified from *S. cerevisiae* were compared to 11677 amino acid sequences consisting of 5880 non-redundant *S. cerevisiae* protein sequences obtained from the National Center for Biotechnology (2009-10-27 release). Randomized versions of each non-redundant protein entry were included in the databases to estimate the false discovery rates (FDR)^47^. All SEQUEST searches were performed with a static modification of +57 Daltons added to cysteine residues to account for carboxamidomethylation, and dynamic searches of +16 Daltons for oxidized methionine. Spectra/peptide matches were filtered using DTASelect/CONTRAST^48^. In this dataset, spectrum/peptide matches only passed filtering if they were at least 7 amino acids in length and fully tryptic. The DeltCn was required to be at least 0.08, with minimum XCorr value of 1.8 for singly-, 2.0 for doubly-, and 3.0 for triply-charged spectra, and a maximum Sp rank of 10. Proteins that were subsets of others were removed using the parsimony option in DTASelect on the proteins detected after merging all runs. Proteins that were identified by the same set of peptides (including at least one peptide unique to such protein group to distinguish between isoforms) were grouped together, and one accession number was arbitrarily considered as representative of each protein group. Quantitation was performed using label-free spectral counting. The number of spectra identified for each protein was used for calculating the distributed normalized spectral abundance factors (dNSAF)^29^. NSAF v7 (an in-house developed software) was used to create the final report on all non-redundant proteins detected across the different runs, estimate false discovery rates (FDR), and calculate their respective distributed Normalized Spectral Abundance Factor (dNSAF) values. The mass spectrometry dataset used in this study has been deposited at https://massive.ucsd.edu/ with the MassIVE ID: MSV000079138.

### Statistical analysis

*INO80 dataset.* First, data generated from the wild-type was compared with control samples. In this case, due to the large number of missing points in the control dataset, we calculated the fold change between the average dNSAF values in wild-type Ino80 and the average dNSAF values in the controls for all detected proteins. All the proteins that were having a fold ratio of four or greater were considered specific proteins. These proteins were next used for the analysis. To address the question of whether any differences were observable between the wild-type and mutant samples, we calculated first the fold changes between the respective purifications using the QSPEC statistical framework (version 1.2.2. QSPEC) using the web submission at http://www.nesvilab.org/qspec.php/. The spectra counts and the length of each protein were used as input for the QSPEC software. Generally, the model is based on a Poisson model with hierarchical Bayesian estimation as described in refs 34 and 49. Proteins with a log2 fold change of −2 or less (i.e. proteins present a significant decrease in the spectra counts in the mutants when compared with the wild-type) were considered for our next analysis. A total of 196 proteins passed the criteria. *Sin3 dataset.* Z-scores and FDR obtained from QSPEC were used to determine the significance change between DMSO- and SAHA-treated samples. Proteins with a Z-score greater than or equal to 2 and a FDR less than or equal to 0.05 in at least one bait were considered significant for the analysis. In a normal distribution, a Z-score of 2 is equivalent to a p-value of 0.02 (p-value < 0.05), a widely used significance threshold.

### Topological data analysis

The input data for TDA are represented in a bait–prey matrix, with each column corresponding to purification of a bait protein and each row corresponding to a prey protein: values are spectral counts for each protein. A network of nodes with edges between them is then created using the TDA approach based Ayasdi 3.0 Cure 3.0 software (AYASDI Inc., Menlo Park CA)^11^,^12^,^50^. Nodes in the network represent clusters of proteins. Nodes in the figures are colored based on the metric PCA1 and PCA2. Two types of parameters are needed to generate a topological analysis: First is a measurement of similarity, called metric, which measures the distance between two points in space (i.e. between rows in the data). Second are lenses, which are real valued functions on the data points. Lenses could come from statistics (mean, max, min), from geometry (centrality, curvature) and machine learning (PCA/SVD, Autoencoders, Isomap). In the next step the data is partitioned. Lenses are used to create overlapping bins in the data set, where the bins are preimages under the lens of an interval. Overlapping families of intervals are used to create overlapping bins in the data. Metrics are used with lenses to construct the Ayasdi 3.0 output. There are two parameters used in defining the bins. One is *resolution*, which determines the number of bins; higher resolution means more bins. The second is *gain*, which determines the degree of overlap of the intervals. Once the bins are constructed, we perform a clustering step on each bin, using single linkage clustering with a fixed heuristic for the choice of the scale parameter. This gives a family of clusters within the data, which may overlap, and we will construct a network with one node for each such cluster, and we connect two nodes if the corresponding clusters contain a data point in common.

For the INO80 yeast dataset, we used correlation metric and two types of lenses (principal and secondary metric singular value decomposition). Resolution and gain were set to 30 and 3.0x eq. for Fig. 2A. In order to determine the structural modularity of the INO80 complex (i.e. generate more bins) we set the resolution to 45 with gain 3.0x eq. (Fig. 2B). In the case of the Sin3-drug network dataset, two types of lenses (Neighborhood Lenses 1 and 2) with norm correlation metric were used. Resolution 20 with gain 3.0x eq. were used to generate Fig. 5A of the entire network and resolution 30 with gain 3.0x eq. were set to generate the modules in Fig. 5B.

Metric equations:

The correlation distance between two points is given by the Pearson correlation and is given by:


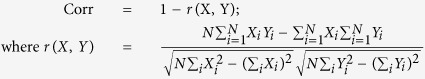


Norm correlation is defined as:

NormCorr = 1 − (X′, Y′) where X′ and Y′ are the variance normalized version of X and Y.

#### Clustering Analysis

In order to gauge the relationship between proteins we applied the hierarchical clustering algorithm using the Ward method and Pearson Correlation as described previously^44^ using INO80 ratio fold changes. We applied k-means clustering to the INO80 ratio fold changes obtained from QSPEC^34^ using Hartigan-Wong algorithm and iter.max = 500000. To determine the best partition of our data we continuously increased the number of clusters. The result showed that the optimal number of clusters was obtained when k=8, after carefully inspecting all the clusters and their silhouette (Supplementary Figure S4). All computations were run using R environment using k-means function (https://stat.ethz.ch/R-manual/R-devel/library/stats/html/kmeans.html) for the partition and daisy function to compute all the pairwise dissimilarities (distances from Euclidean) between observations in the data set for the silhouette.

## Additional Information

**How to cite this article:** Sardiu, M. E. *et al*. Identification of Topological Network Modules in Perturbed Protein Interaction Networks. *Sci. Rep.* **7**, 43845; doi: 10.1038/srep43845 (2017).

**Publisher's note:** Springer Nature remains neutral with regard to jurisdictional claims in published maps and institutional affiliations.

## Supplementary Material

Supplementary Information

Supplementary Dataset 1

Supplementary Dataset 2

Supplementary Dataset 3

Supplementary Dataset 4

Supplementary Dataset 5

Supplementary Dataset 6
