# Supplementary material for: Identification of Topological Network Modules in Perturbed Protein Interaction Networks
Source: Sci Rep. 2017 Mar 8;7:43845. doi: 10.1038/srep43845 (PMC5341041; doi:10.1038/srep43845)
Supplement: Supplementary Information [file srep43845-s1.pdf]

Supplementary Information For:

Identification of Topological Network Modules in Perturbed Protein Interaction Networks

Mihaela E. Sardiu[1#], Joshua M. Gilmore[1#], Brad Groppe[2], Laurence Florens[1], and Michael P. Washburn[1,3‡]

[1]Stowers Institute for Medical Research, Kansas City, MO 64110 U.S.A.
[3] Department of Pathology and Laboratory Medicine, The University of Kansas Medical Center, 3901 Rainbow Boulevard, Kansas City, Kansas 66160, USA

# These authors contributed equally to this work
To whom correspondence should be addressed:
Email: mpw@stowers.org

Supplementary Figure S1: Relative abundance of INO80 subunits

The relative levels of each of the 15 subunits of the ION80 complex were plotted as dNSAF values. (A-K) dNSAF values of the complexes recovered from the 11 deletion strains were compared against their respective wild-type affinity purifications. In each of these dNSAF plots, the TAP-tagged bait protein is indicated on the x-axis within a solid color box, while the deleted gene is within a grey box. The proteins that are lost or with significant decrease in levels in the deletion strains are indicated by colored boxed. (L-O) dNSAF values of the INO80 complexes affinity purified using baits localized to different modules. In all plots, averages ± standard deviations are shown (see detailed results for individual analyses in Supplemental Tables 1 and 2).

Supplementary Figure S2. Graphical summary of the distribution of the log2(fold_change) of proteins in six mutants in TNM 1 (A) and in TNM 2 (B) of the INO80 topological network. Deletion of subunits located within the same structural module are illustrated by the same color and have close medians as shown in the parallel boxplots.

Supplementary Figure S3. Unsupervised hierarchical clustering on the prey proteins of whose abundance changed from wild-type to mutants. Qspec ratio was used as input for the cluster analysis. Subunits of the INO80 complex are colored as in Figure1. Proteins detected in the TNM 2 are colored here in purple.

Supplementary Figure S4. Silhouette plot. K-means clustering on the Qspec ratio on prey proteins whose abundance changed between wild-type and mutants. Hartigan-Wong algorithm and iter.max=50000 were used for the analysis. The optimum number of k-means clusters was determined to be 8 based on the silhouette plot. Average silhouette width was 0.42 for k=8.

Supplementary Table S1. MudPIT analysis and label-free quantitation of the proteins detected in the

***S. cerevisiae* INO80 network.A.** Subunits of the INO80 complex detected in affinity purifications of TAP-tagged INO80 subunits from S. cerevisiae. Proteins abundance represented by dNSAF values are included in this table. **B.** Proteins detected in affinity purifications of TAP-tagged INO80 subunits from wild-type and deletion strains of S. cerevisiae (before contaminant extraction).

Supplementary Table S2. Statistical steps to define the input for Toplogical Data Analysis of the *S. cerevisiae* INO80 deletion network. A. QSPEC statistical results between negative controls and wild-type INO80-TAP purificationns. Proteins that passed a filter cutoff (i.e. a ratio greater than 4) are considered significant proteins for our analysis, while all others are considered non-specific contaminants and removed. **B.** QSPEC statistical results between WT INO80-TAP and deletion mutants after contaminant extraction. **C.** Proteins with a Log2(Fold-Change) lesser or equal to -2 in at least one deletion analysis are used as input for Topological Data Analysis. These Log2(FoldChange) values are used to build Figure 3A.

Supplementary Table S3. Topological Network Modules defined by Topological Data Analysis of the INO80 deletion network.. LogFoldChanges for each protein in the module are too reported in this table.

Supplementary Table S4. Clustered proteins based on the k-means. Proteins are separated in 8 clusters. Cluster numbers are included in the table. Proteins of the INO80 complex were colored in red. The 149 proteins used are the same as used in TDA and hierarchical clustering

Supplementary Table S5. Proteins detected in the Sin3 wild-type data after contaminant extraction. Qspec results on proteins detected in wild-type baits and baits treated with SAHA. Only proteins with a significant Z-score (i.e. Z-score >=2) and a FDR less than equal to 0.05 are included in this table.

Supplementary Table S6. Identified modules in the Sin3 perturbed network by Topological Data Analysis (TDA). Z-statistics of each protein in each bait in a module are reported in this table.

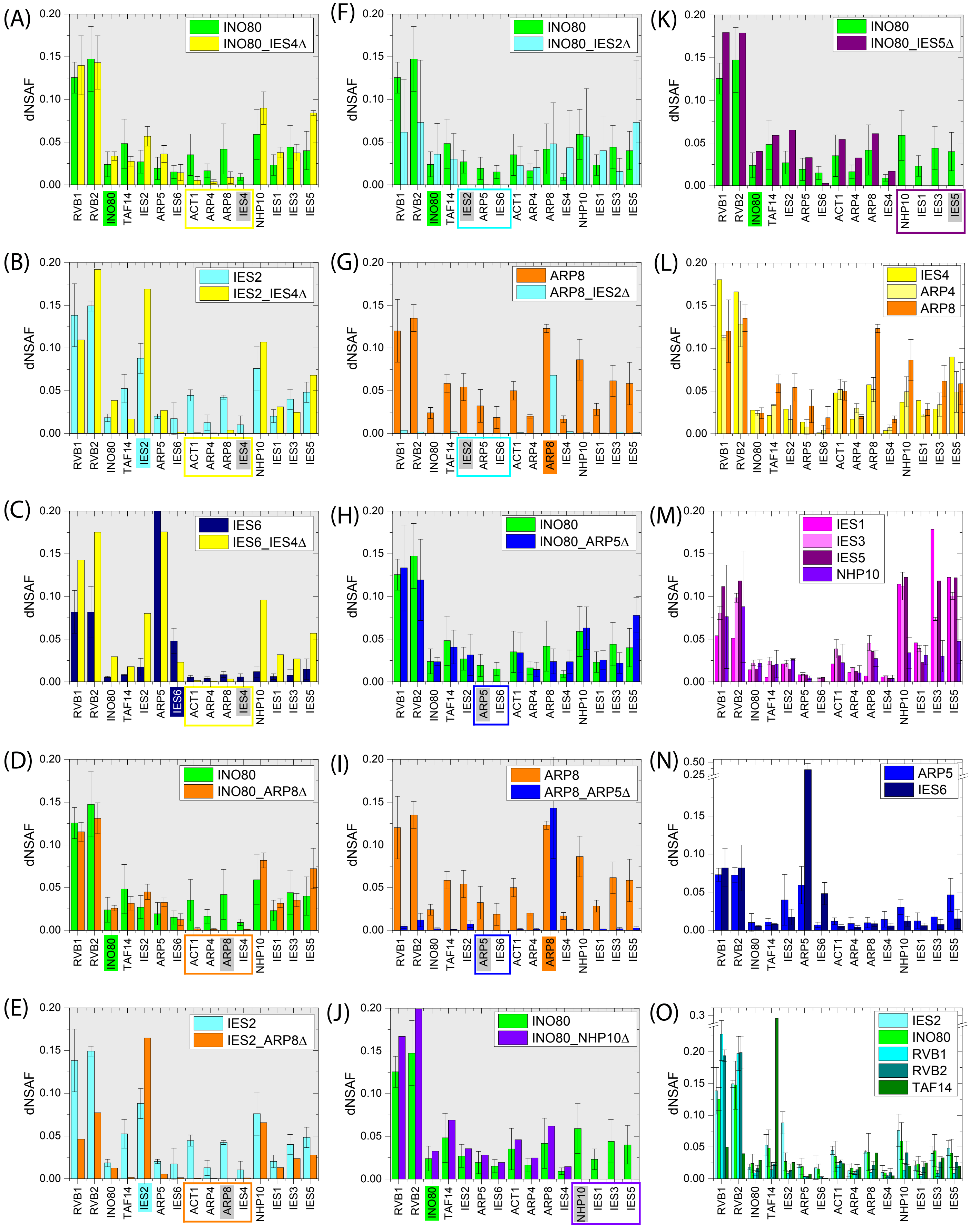

Supplementary Figure S1. Sardiu et al.

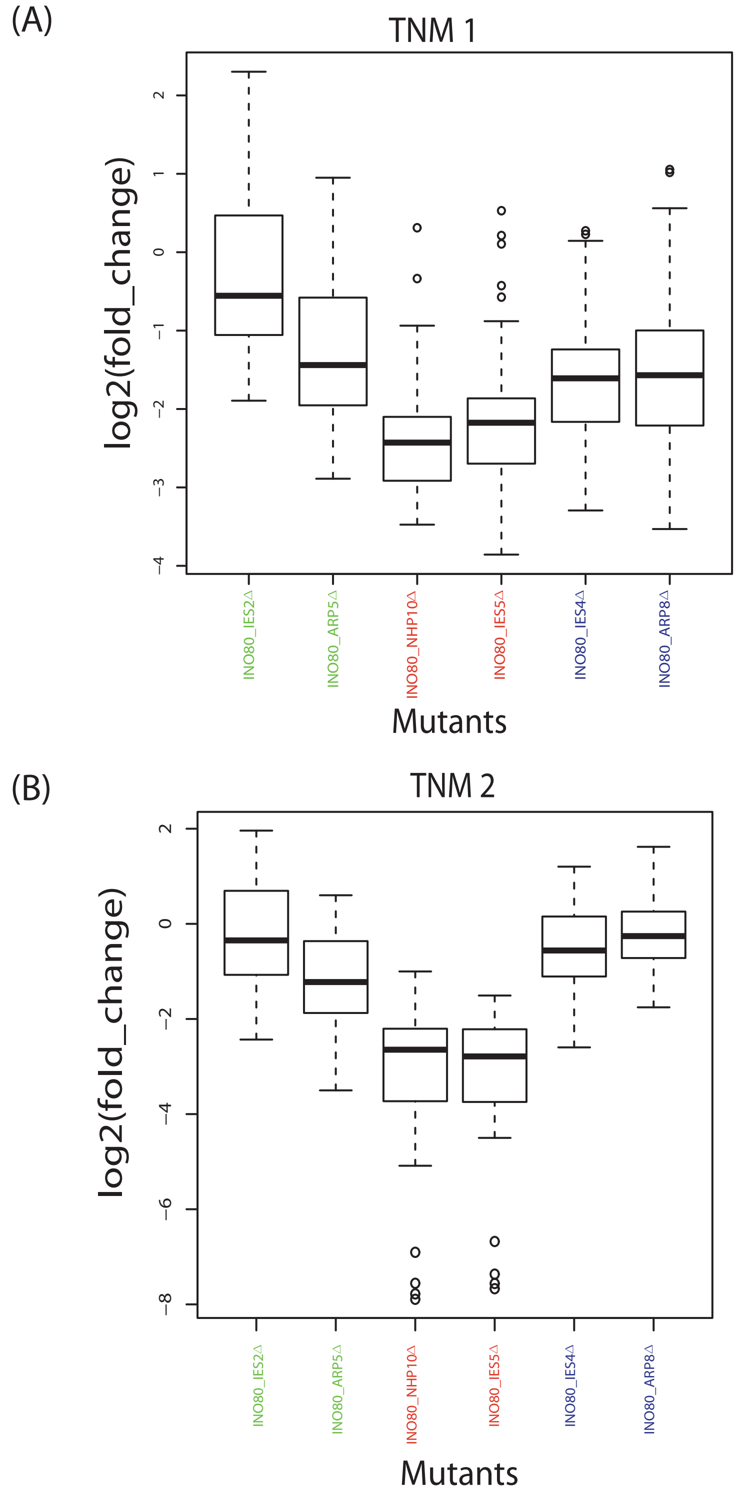

Supplementary Figure S2. Sardiu et al.

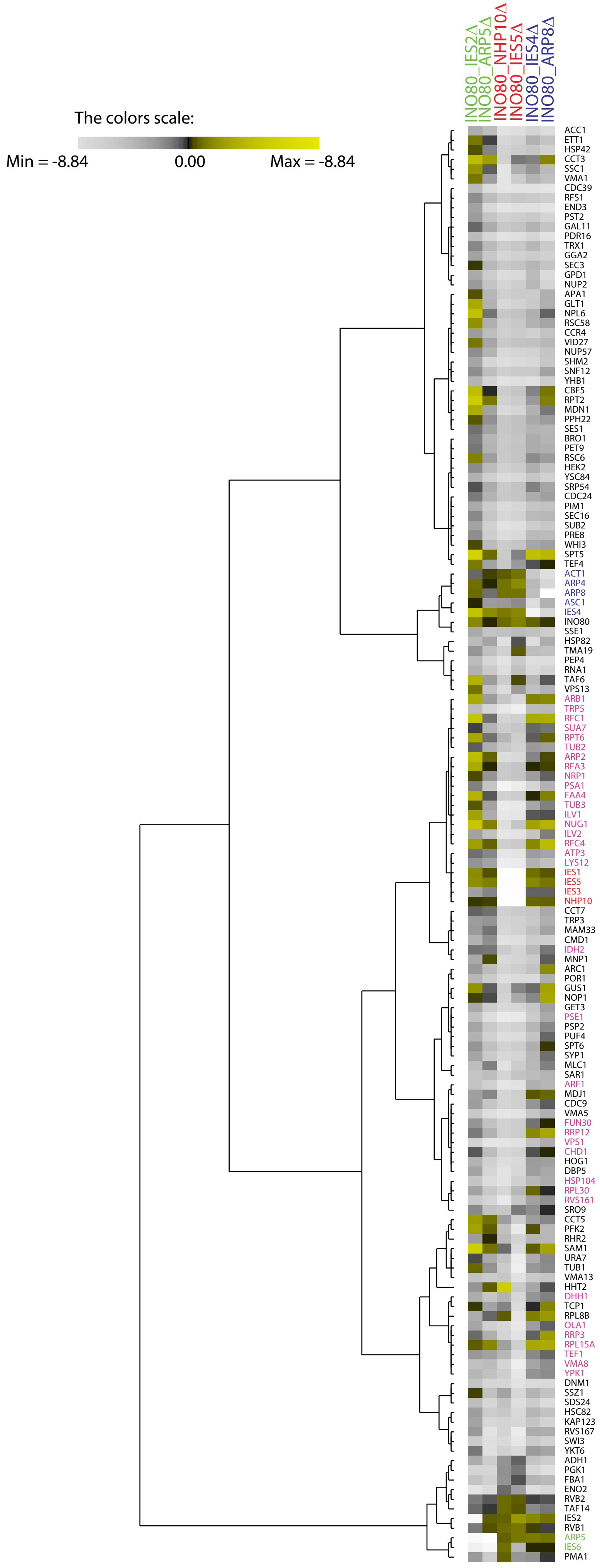

Supplementary Figure S3. Sardiu et al.

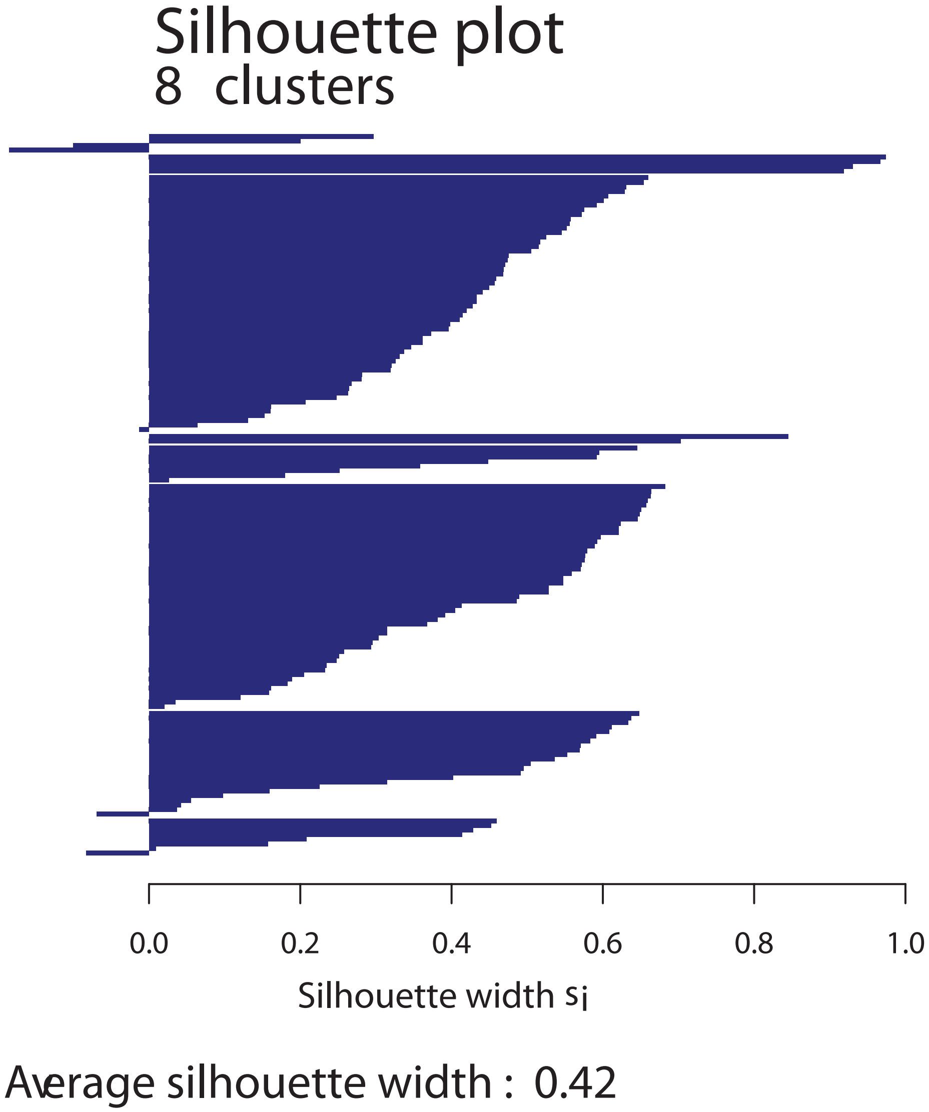

Supplementary Figure S4. Sardiu et al.
